# The Potential Effects of Dielectric Barrier Discharge Plasma on the Extraction Efficiency of Bioactive Compounds in *Radix Paeoniae Alba*

**DOI:** 10.3389/fnut.2021.735742

**Published:** 2021-10-26

**Authors:** Tao Jin, Zhenghua Zhou, Jian Zhou, Wenchong Ouyang, Zhengwei Wu

**Affiliations:** ^1^School of Nuclear Science and Technology, University of Science and Technology of China, Hefei, China; ^2^Anhui Academy of Medical Sciences, Hefei, China; ^3^Key Laboratory of Geospace Environment, Chinese Academy of Sciences, Hefei, China

**Keywords:** *radix paeoniae alba* (RPA), dielectric barrier discharge plasma, extraction efficiency (EE), high performance liquid chromatography (HPLC), ultraviolet spectrophotometer

## Abstract

*Radix paeoniae alba* (RPA) is a kind of herbal medicine of traditional Chinese medicine (TCM) that is widely used for the treatment of liver diseases and rheumatoid arthritis in clinical practice. As a result of the low extraction efficiency of RPA by the conventional method, many patients are given high dosages. In this study, four exposure doses of dielectric barrier discharge (DBD) plasma (0, 60, 120, and 180 s) were applied to modify the extraction efficiency of paeoniflorin, benzoylpaeoniflorin, tannic acid, gallic acid, 2′-hydroxy-4′-methoxyacetophenone, and polysaccharide in RPA. Finally, the application of plasma for 180 s exhibited a 24.6% and 12.0% (*p* < 0.001) increase of tannic acid and polysaccharide contents, however, a 2.1% (*p* < 0.05) and 5.4% (*p* < 0.001) reduction of paeoniflorin and gallic acid composition, respectively, and no significant difference (*p* > 0.05) in results obtained from benzoylpaeoniflorin and 2′-hydroxy-4′-methoxyacetophenone contents. Our results of scanning electron microscopy (SEM), automatic specific surface area and pore analyzer, Fourier transform infrared spectroscopy (FTIR), X-ray photoelectron spectroscopy (XPS), and thermal gravimetric analysis (TGA) indicated that DBD plasma can etch the surface and undergo graft polymerization by reactive species thereby changing the water/oil holding capacity and eventually changing the extraction efficiency of bioactive compounds in RPA. Overall, our observations provide a scientific foundation for modifying the extraction efficiency of bioactive ingredients related to the pharmacological activities of RPA.

## Introduction

*Radix Paeoniae Alba* (RPA) is the sunlight dried root of *Paeonia Lactiflora Pall*. without bark (1). Paeoniflorin, benzoylpaeoniflorin, tannic acid, gallic acid, 2′-hydroxy-4′-methoxyacetophenone, and polysaccharide are considered the main bioactive components in RPA, which can be used as a medicinal herb in traditional Chinese medicine (TCM) (2–5). In modern clinical practices, RPA exerts remarkable abilities to downregulate the mRNA and protein expression of inducible nitric oxide synthase (iNOS) and suppress interleukin-6 (IL-6) and tumor necrosis factor alpha (TNF-α) release (6, 7). Therefore, RPA has been diffusely applied to treat rheumatoid arthritis (8), cardiac diseases (9), hepatitis (10), dysmenorrhea (11), and other inflammation-related disorders (12), due to its pharmacological benefits in multiple cells and tissues. On the other hand, RPA also presents a potential herb-herb interaction to stimulate or inhibit the nephrotoxicity and neurotoxicity side effects of *Semen Strychni* (maqianzi in Chinese) or *Aconiti Lateralis Radix Praeparata* (fuzi in Chinese) (13, 14). Though RPA shows various properties to treat clinical diseases, the extraction efficiency and bioavailability of bioactive compounds in RPA by conventional methods is low. In addition, the long period to oral administration of RPA decoctions has resulted in higher economic and psychological burden on patients. Therefore, it is meaningful to find an effective, safe, and simple strategy to improve the extraction efficiency of bioactive substances in RPA.

Plasma is a kind of ionized gas containing negative and positive ions, free radicals, neutral particles, and electronic and UV light (15, 16). Low temperature plasma (LTP), known as non-Thermal plasma, can operate at ambient temperature and atmospheric pressure. LTP has been widely applied to materials decoration and drugs efficiency due to its non-Thermal, non-Toxic, and green characteristics (17–21). In recent years, the results of Bao et al. showed that high voltage (60 kV) atmospheric cold plasma (HVACP) treatment for 15 min can increase the yield of phenolic extracts of grape pomace by 10.9–22.8%, which also showed an improved antioxidant capacity (16.7–34.7%) (22). Furthermore, the study of Rashid et al. has proved that HVACP treatment with air at 80 kV for 30 min resulted in increased galactomannan extraction yields by 122% from soaked seeds and 67% from dry seeds (23). Thus, taking account of the enhancement properties of HVACP on the nutrition value of fruit residue, plant seeds, and other medicines, we aimed to apply DBD plasma, one of the HVACP systems, to modify the extraction efficiency of bioactive compounds in RPA, including paeoniflorin, benzoylpaeoniflorin, tannic acid, gallic acid, 2′-hydroxy-4′-methoxyacetophenone, and polysaccharide. In this study, the output voltage, current, and frequency of the DBD plasma are around 8 kV, 40 mA, and 20 kHz, respectively. RPA samples were exposed to four doses of DBD plasma (0, 60, 120, and 180 s), and the contents of the six ingredients in all groups were measured. To explore the underlying mechanisms, assays of water/oil holding capacity (W/OHC), scanning electron microscope (SEM), automatic specific surface area and pore analyzer, Fourier transform infrared spectrometer (FTIR), X-ray photoelectron spectroscopy (XPS), and thermal gravimetric analyzer (TGA) were carried out.

## Materials and Methods

### Materials and Reagents

RPA was purchased from Yonggang Decoction Piece Factory Co., Ltd. (Bozhou, China). The standard substances including paeoniflorin, benzoylpaeoniflorin, gallic acid, tannic acid, 2′-hydroxy-4′-mehoxyacetophenone, and D-(+)-glucose were HPLC grade and bought from Shanghai Macklin Biochemical Co., Ltd (Shanghai, China). The water used in this study was deionized and prepared by the PSDK-C system (Beijing, China).

### Samples

**Figure 1:**
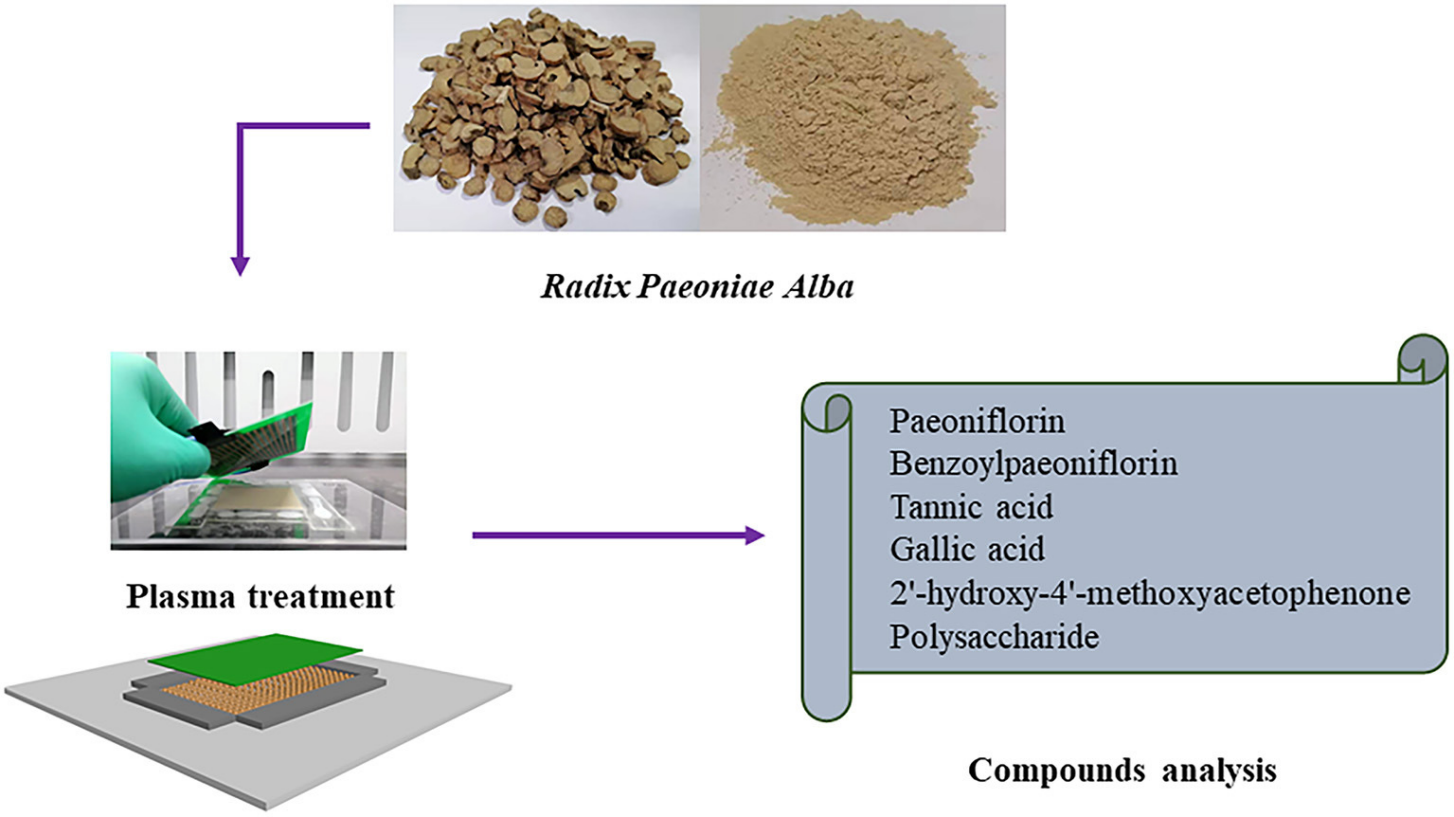
Schematic diagram of experimental design and DBD plasma treatment.

As shown in Figure 1, the sunlight-dried RPA pieces were crushed into powder and sieved (120 mesh). Afterwards the handmade platform (7.5 × 8.0 × 0.1 cm) was filled with RPA and the DBD board was placed at 0.1 cm height from the platform. The RPA samples were treated with DBD plasma at three doses of 60 s, 120 s, and 180 s and recorded as DP-60, DP-120, and DP-180, respectively. The untreated RPA, also regarded as control, was coded as DP-0.

### Bioactive Compounds Measurement

As explained above, the extracted content of each bioactive compound in RPA was measured according to the conventional methods (include high performance liquid chromatography and ultraviolet spectrophotometer) in *Chinese Pharmacopeia 2020*, with some modifications. **Paeoniflorin:** 0.5 g of RPA was immersed in 80 mL of methanol solution, assisted by ultrasonic extraction for 30 min, and cooled to room temperature. Then the extraction was supplemented to 100 mL with methanol. Finally, the combined solution was filtered with a 0.45-μm microporous membrane. The extraction was separated on a Shim-pack VP-ODS C18 chromatographic column (4.6 × 150 mm, 5 μm; Shimadzu, Japan). The mobile phase was acetonitrile-0.1% phosphate buffer (PBS) at a stable flow rate of 1.0 mL/min and a split ratio of 14:86. The temperature of the column was controlled at 30°C and the injection samples volume was set at 10 μL. The absorbance values were obtained at 230 nm and calculated using Chromeleon (c) Dionex software. **Benzoylpaeoniflorin and 2′-hydroxy-4′-methoxyacetophenone:** The extractions were prepared by the same procedures as paeoniflorin, but the mobile phase was changed to methanol-water (45:55). Accompanied by the same flow rate, column temperature, and injection volume, the contents of 2′-hydroxy-4′-methoxyacetophenone and benzoylpaeoniflorin were detected at 230 and 274 nm, respectively. **Tannic acid and gallic acid:** 0.1 g of RPA was immersed in 100 mL of deionized water for 30 min, shaken in a constant temperature bath at 60°C for another 30 min, and cooled to room temperature. Finally, the absorbance values of tannic acid and gallic acid were obtained at 276 and 263 nm, respectively, and the contents of the two bioactive compounds were calculated using standard curves (Y_tannicacid_ = 0.0398 X−0.00865, R = 0.9999; Y_gallicacid_ = 0.0451 X +0.00297, R = 0.9998). **Polysaccharide:** 0.5 g of RPA was immersed in 50 mL of deionized water, accompanied by heating reflux extraction for 2 h. The extracted solution was centrifuged at 7,000 rpm for 10 min and 2 mL of supernatant was mixed with 10 mL of absolute ethanol. After being centrifuged at 7,000 rpm for 20 min, the sediment was washed with 8 mL of 80% ethanol twice. Finally, the washed sediment was dissolved with 50 mL of hot deionized water and the absorbance value and content were obtained at 488 nm (Y_polysaccharide_ = 0.006289 X +0.0447, R = 0.9999). All assays were conducted at least three times and the values were recorded as mean ± standard deviations (std) mg/g. To evaluate the effects of DBD plasma treatment on the extraction efficiency of RPA, the percentage of content of each compound was obtained by the following equation,


(1) $$\begin{array}{l} The\;percentage\;of\;content\;\left(\%\right)=\frac{m_{p}-{\text{m}}_{0}}{{\text{m}}_{0}}\times 100 \end{array}$$

where *m*_*p*_ and m_0_ represent the content of DBD plasma-treated and untreated RPA samples, respectively.

### Water/Oil Holding Capacity

The water and oil holding capacities of RPA were studied according to Nawaz et al., with some modifications (24). A total of 0.2 g of RPA powder was mixed with 5 mL of water or oil (Luhua peanut oil, Shandong Luhua Group Co. Ltd, Laiyang, Shandong, China), the value was calculated by the following equation after being vortexed for 1 min and left to stand in the dark for 24 h,


(2) $$\begin{array}{l} Water/Oil\;holding\;capacity\;\left(\%\right)=\frac{m_{1}-{\text{m}}_{0}}{{\text{m}}_{0}}\times 100 \end{array}$$

where *m*_1_ and m_0_ represent the weight of soaked and dried RPA samples, respectively.

### Characteristic Analysis

The surface morphology of the four RPA samples was observed using a Scanning Electron Microscope (GeminiSEM 500, Germany), and the specific surface area and pore change were determined by a Tristar II 3020M analyzer (Micromeritics, USA). The FTIR spectra of the four RPA samples were recorded on a Fourier transform infrared spectrometer (Thermo Nicolet 8,700, USA). Each sample was blended with KBr at a 1:20 ratio and scanned from 4,000 to 400 cm^−1^ wavenumbers. The XPS spectra were recorded on a Thermo ESCALAB250Xi spectrometer (Thermo Fisher Scientific Co. Ltd, UK) with an excitation source of monochromatized Al Kα (hv = 1486.6 eV) and a pass energy of 30 eV. The values of binding energies were calibrated with the C 1s peak of contaminant carbon at 284.80 eV. The thermostability of RPA was characterized using a thermal gravimetric analyzer (TGA Q5000iR, USA) at a heating procedure of 5°C/min from 25 to 600°C under a nitrogen atmosphere.

### Statistical Analysis

All assays were conducted at least three time with the values recorded as mean ± std. Significance of differences in data was determined by IBM SPSS Statistics 21 (International Business Machines Corporation, USA), and *p* < 0.05, *p* < 0.01, and *p* < 0.001 represented a significant, highly significant, and extremely significant difference, respectively, as compared with control (DP-0). All figures were plotted by Origin 8.5 and the XPS results were analyzed using XPSPEAK4.1 (Raymund W.M. Kwok, The Chinese University of Hong Kong, Hong Kong, China).

## Results

### Contents of the Six Bioactive Compounds in RPA Extraction

As shown in Table 1, the paeoniflorin content of RPA in the DP-180 group exerted a significant reduction (*p* < 0.05) of 2.1% as compared with the DP-0 group, while the content in the DP-60 and DP-120 groups showed no significant (*p* > 0.05) changes. The benzoylpaeoniflorin and 2′-hydroxy-4′-methoxyacetophenone contents of RPA in the DP-60, DP-120, and DP-180 groups all showed a non-Significantly difference (*p* > 0.05) as compared with the DP-0 group. In the DP-60 and DP-120 groups, tannic acid and polysaccharide contents had non-Significant (*p* > 0.05) changes as compared with the DP-0 group, however, the content of that in the DP-180 group presented a highly significant (*p* < 0.001) increase of 24.6 and 12.0%, respectively. Finally, the gallic acid contents in the DP-60 and DP-120 groups showed a highly significant (*p* < 0.01) reduction of 3.5 and 3.4% respectively, while the content of that in the DP-180 group exhibited a highly significant (*p* < 0.001) reduction of 5.4% as compared with the DP-0 group.

**Table 1:** Six bioactive compound contents in the extraction of RPA: DP-0, DP-60, DP-120, and DP-180 groups.

**Content (mg/g)**	**Plasma Treatment**			
	**DP-0**	**DP-60**	**DP-120**	**DP-180**
Paeoniflorin	12.80 ± 0.18	13.00 ± 0.12^#^	12.68 ± 0.06^#^	12.53 ± 0.12^*^↓(2.1%)
Benzoylpaeoniflorin	0.48 ± 0.02	0.50 ± 0.01^#^	0.47 ± 0.01^#^	0.49 ± 0.03^#^
Tannic acid	9.26 ± 0.42	9.80 ± 0.17^#^	9.60 ± 0.17^#^	11.54 ± 0.59^***^↑(24.6%)
Gallic acid	7.96 ± 0.1	7.68 ± 0.14^**^↓(3.5%)	7.69 ± 0.04^**^↓(3.4%)	7.53 ± 0.06^***^↓(5.4%)
2′-hydroxy-4′-methoxyacetophenone	0.11 ± 0.00	0.11 ± 0.00^#^	0.11 ± 0.00^#^	0.11 ± 0.00^#^
Polysaccharide	148.23 ± 5.24	151.40 ± 6.06^#^	150.03 ± 3.16^#^	166.08 ± 4.01^***^↑(12.0%)

# *p > 0.05*;

* *p < 0.05*;

** *p < 0.01*;

*** *p < 0.001 vs the DP-0 group*.

*The symbols of ↓ and ↑ indicated the bioactive compounds contents in DP-60, DP-120, and DP-180 groups were decrease or increase respectively as compared with that in DP-0. Bold values displayed the decrement and increment of extraction efficiency as compared with control*.

### Water/Oil Holding Capacity

**Figure 2:**
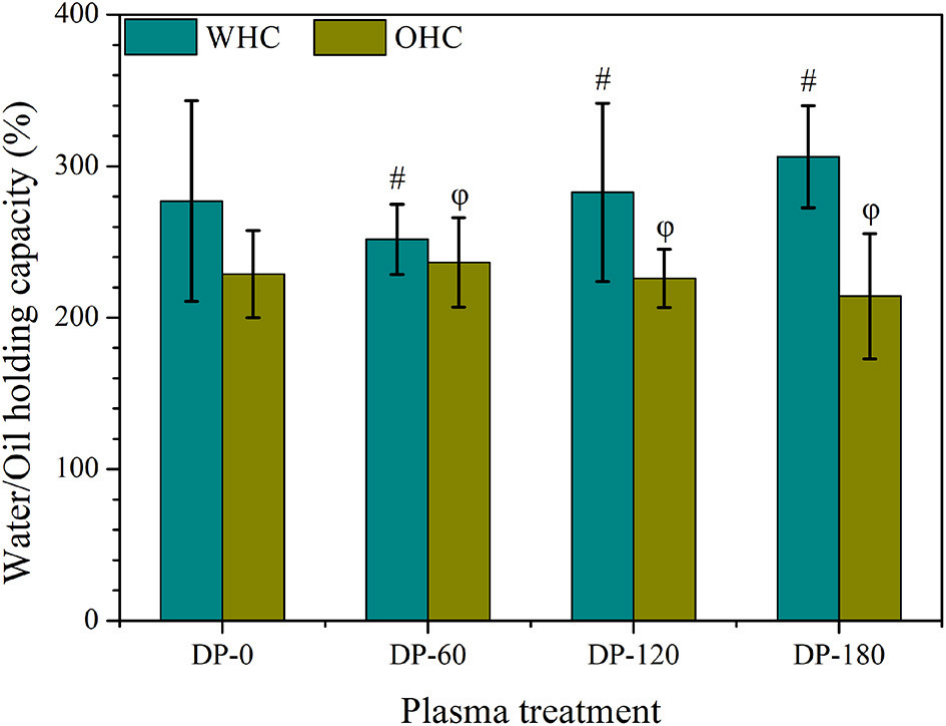
The water and oil holding capacity of RPA in DP-0, DP-60, DP-120, and DP-180 groups. # and ϕ represents *p* > 0.05 vs. the DP-0 group.

As shown in Figure 2, non-Significant (*p* > 0.05) changes were observed in the water and oil holding capacity between the DP-0 group with DP-60, DP-120, and DP-180 groups, while the water holding capacity of RPA in the DP-120 and DP-180 groups further improved as compared with the DP-0 group.

### Scanning Electron Microscopy

**Figure 3:**
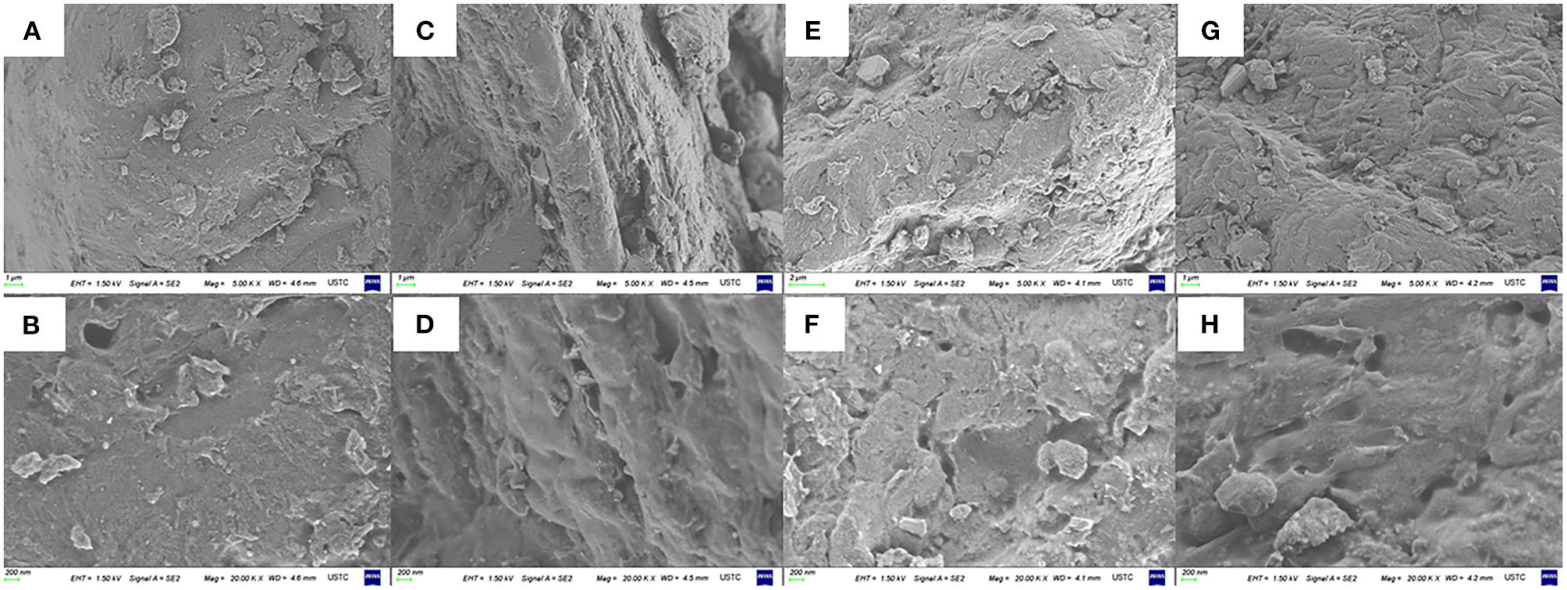
The surface morphologies of RPA at 5.00 K × and 20.00 K × magnifications in the DP-0 **(A,B)**, DP-60 **(C,D)**, DP-120 **(E,F)**, and DP-180 **(G,H)** groups.

As shown in Figures 3A,B, the untreated RPA had a rough surface and various small particles. However, it can be seen in Figures 3C,D, the RPA in the DP-60 group had some shallow gaps and a smoother plane than the DP-0 group. In the Figures 3E,F, the surface morphologies of RPA in the DP-120 group was obviously scratched and had small pores, while the RPA in the DP-180 group showed intensive cracks and smaller pores (Figures 3G,H).

### Automatic Specific Surface Area and Pore Analysis

**Figure 4:**
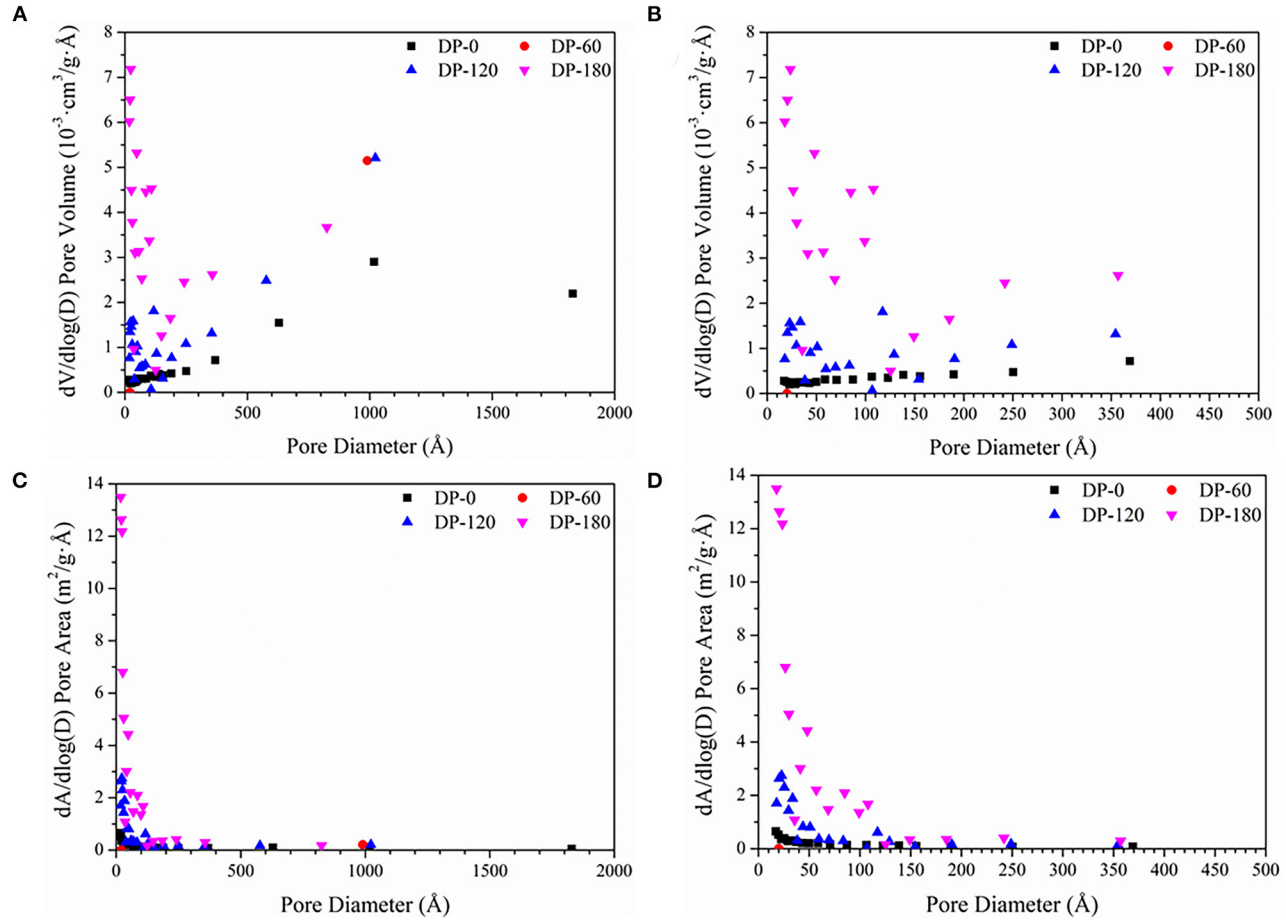
Distribution of pore diameter as functions with dV/dlog(D) pore volume **(A,B)** and dA/dlog(D) pore area **(C,D)** respectively. **(A,C)** are the pore diameter distribution in the full range of 0–2000 Å (Å = 0.1 nm), while **(B,D)** are the pore diameter distribution of mesopores (0–500 Å).

As can be seen in Table 2, the BET specific surface area of RPA in the DP-0 group was 0.4634 m^2^/g. The area of that in the DP-60 group reduced to 0.0421 m^2^/g, while it increased to 1.3328 m^2^/g in the DP-120 group. With a longer plasma treatment time, the area obtained a significant improvement of 7.7209 m^2^/g in the DP-180 group. As shown in Figures 4A,B, the pore diameter of RPA in the DP-0 group was located in the range of 0-2000 Å and most in 0-500 Å, while each pore occupied a lower pore volume. In the DP-60 group, the pore diameter of RPA was reduced to two ranges around 1,000 and 20 Å, while the proportion of 1000 Å particles increased and the 20 Å grains decreased. In the DP-120 and DP-180 groups, the RPA particles were further scaled into smaller size, and the particles in the DP-120 group were uniformly distributed in the range of 0–400 Å at a lower rate. Contrarily, the particles in the DP-180 group were intensively strewn in 0–150 Å and presented a higher pore volume as compared with the DP-120 group. It can be seen in Figures 4C,D, the RPA in the DP-0 group was most distributed in the range of 0–1000 Å and showed a low pore area, while the particles in the DP-60 group displayed a much lower pore area in the same range. In agreement with the results of pore volume, the RPA in the DP-120 and DP-180 groups was densely distributed in the range of 0–150 Å and showed a higher pore area, and the RPA in the DP-180 presented an extremely higher pore area than the DP-120 group.

**Table 2:** BET specific surface area of RPA in the DP-0, DP-60, DP-120, and DP-180 groups.

**Sample**	**BET specific surface area (m^2^/g)**
DP-0	0.4634
DP-60	0.0421
DP-120	1.3328
DP-180	7.7209

### Fourier Transform Infrared Spectrometry

**Figure 5:**
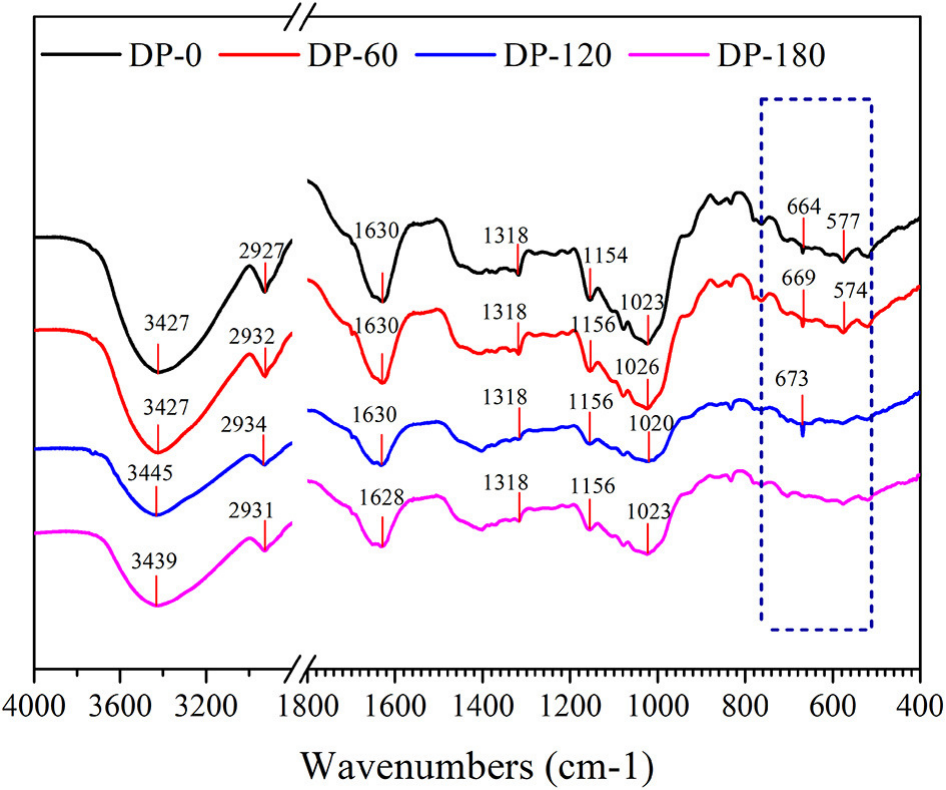
The FTIR spectra (4,000–400 cm^−1^) of RPA in the DP-0, DP-60, DP-120, and DP-180 groups.

As shown in Figure 5, the infrared spectra of RPA in the DP-0, DP-60, DP-120, and DP-180 groups were obtained. The obvious peak around 3,440 cm^−1^ was related to the stretching vibration of the -OH group in the polymer. A peak near to 2,930 cm^−1^ belonged to the asymmetric stretching vibrations of the -CH group on the benzene ring. The peak at 1,630 cm^−1^ was assigned to the stretching vibrations of -C=O in the aromatic ring framework. The stronger peak in the wavenumber of 1,318 cm^−1^ represented the CaC_2_O4 molecules. The peaks around 1,150 cm^−1^ and 1,020 cm^−1^ displayed the characteristic spectra of ether bond (=C-O-C/C-O-C). The peaks in the range of 700–500 cm^−1^ in the four RPA samples were mainly attributed to the external bending vibration of the C-H groups connected with the benzene ring.

### X-Ray Photoelectron Spectroscopy

**Figure 6:**
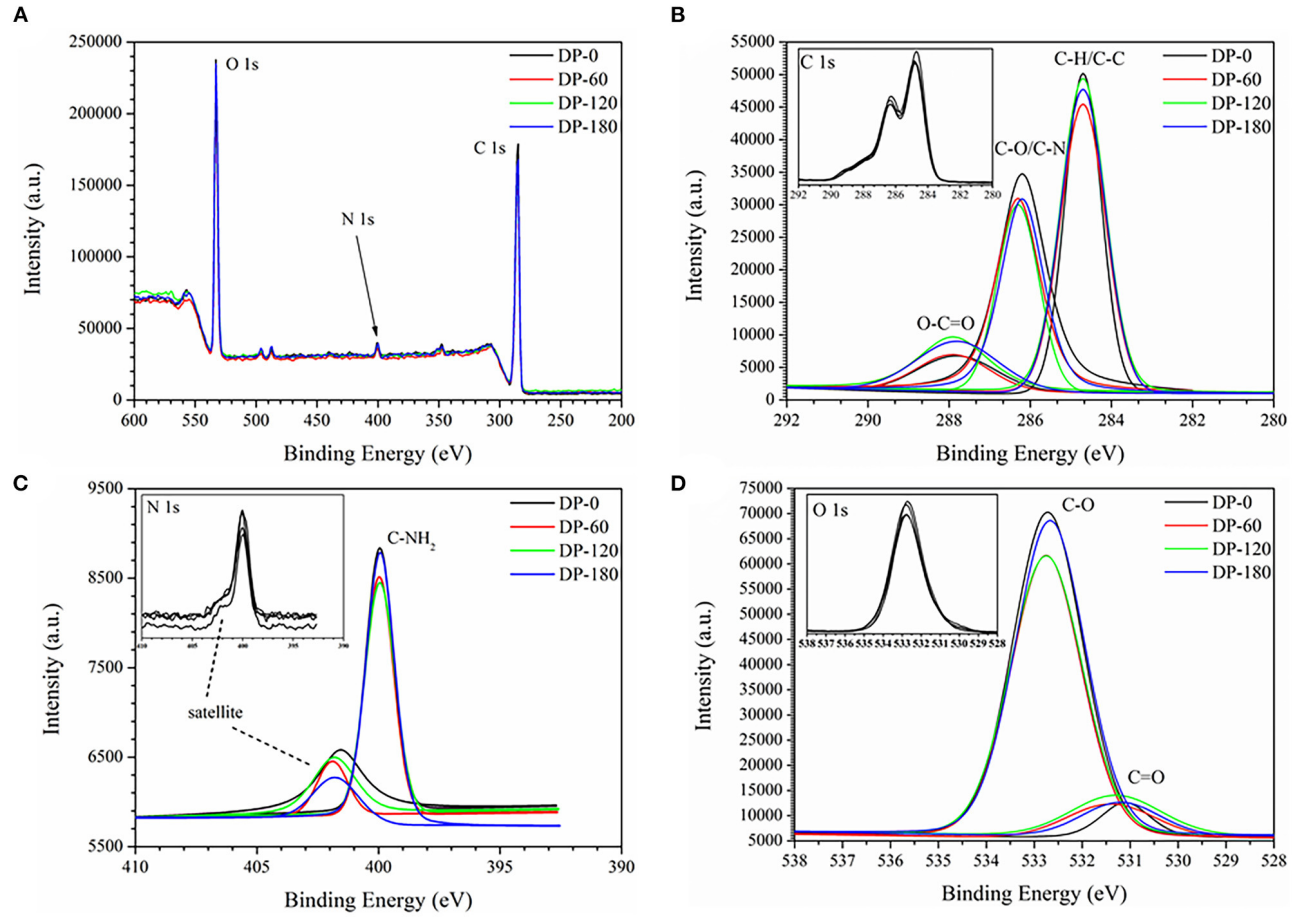
The XPS survey spectra **(A)** and C 1s **(B)**, N 1s **(C)**, and O 1s **(D)** spectra of RPA in the DP-0, DP-60, DP-120, and DP-180 groups.

As shown in Figure 6A, there were three stronger peaks around 285, 400, and 530 eV and they were mainly attributed as the binding energy of C 1s, N 1s and O 1s, respectively. The XPS spectra were calibrated with the C 1s peak of contaminant carbon at 284.8 eV. Figure 6B shows three standard peaks of C 1s around 284.7, 286.2, and 288 eV which were attributed as the binding energy of C-H/C-C, C-O/C-N, and O-C=O, respectively. It also can be seen that three peaks showed a small shift of 0.5–1.0 eV to left in the ranges of 286.2–286.3 eV and 287.9–288.4 eV. As compared with the DP-0 group, the peak intensity of O-C=O was increased with the increment of treatment time from 60 to 180 s. Figure 6C showed a normal peak near 400 eV which was related to the binding energy of C-NH_2_, and the intensity also presented the same increasing trend with plasma exposure duration. Figure 6D shows two obvious peaks at 531.25 and 532.75 eV corresponding to C=O and C-O, respectively. The intensity of C=O of RPA in the DP-60, DP-120, and DP-180 groups was stronger than that in the DP-0 group with an increasing trend over plasma treatment time, while the intensity of C-O in the DP-60, DP-120, and DP-180 groups was weaker than in the DP-0 group. As shown in Table 3, the oxygen atom content of RPA in the DP-60, DP-120, and DP-180 groups was 27.09, 27.27, and 27.61%, respectively, which was higher than the DP-0 group of 26.72%.

**Table 3:** Relative atomic composition (C 1s, O 1s, and N 1s) for the four RPA samples in DP-0, DP-60, DP-120, and DP-180 groups.

**Sample**	**Atomic composition (%)**			**Ratio**
	**C 1s**	**O 1s**	**N 1s**	**O/C**
DP-0	71.28	26.72	2	0.375
DP-60	70.89	27.09	2.03	0.382
DP-120	70.73	27.27	2	0.386
DP-180	70.43	27.61	1.96	0.392

### Thermal Gravimetric Analysis

**Figure 7:**
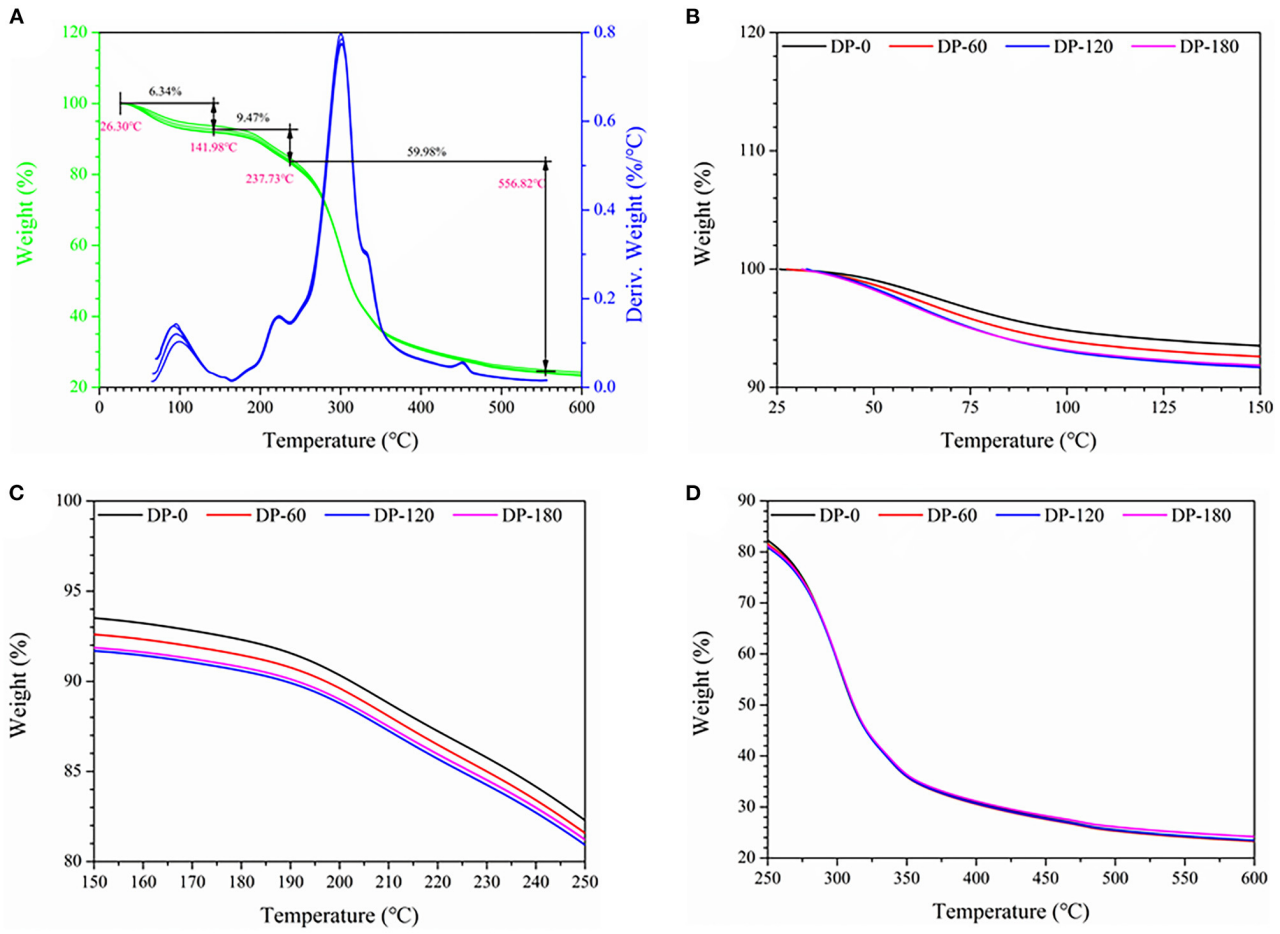
The thermal gravimetric curves of RPA in the DP-0, DP-60, DP-120, and DP-180 groups: **(A)** (25–400 °C), **(B)** (25–150 °C), **(C)** (150–250 °C), and **(D)** (250–600 °C).

The TG and DTG characteristic curves of the four RPA samples are shown in Figure 7A. The four RPA samples displayed similar weight loss regions in the range of 26.30–141.98°C (region I; Figure 7B), 141.98–237.73°C (region II; Figure 7C), and 237.73–556.82°C (region III; Figure 7D), and presented about 6.34%, 9.47%, and 59.98% of weight loss, respectively. In both region I and region II, the weight loss of the RPA samples in the DP-60, DP-120, and DP-180 groups was further decreased as compared with the DP-0 group and the decreased value was related to the plasma treatment time.

## Discussion

In this work, a DBD plasma device was utilized to modify the extraction efficiency of bioactive compounds in RPA due to its powerful reactive oxygen and nitrogen particles (RONS) (25–27). To understand the underlying mechanism, measurement assays of bioactive components were conducted to state the positive and negative effects of DBD plasma on RPA. The W/OHC, SEM, and specific surface area and pore analysis were performed to reveal the microstructural changes on RPA by plasma exposure. Furthermore, the FTIR and XPS assays were operated to explore how the plasma treatment modified the structural of RPA. The TGA experiment aimed to further study the physicochemical changes of RPA.

As expected, the contents of tannic acid and polysaccharide in RPA showed a highly significant improvement in the DP-180 group (Table 1), while paeoniflorin and gallic acid displayed fewer changes as compared with control. The results agreed with the W/OHC assays (Figure 2), in which the WHC of RPA in the DP-180 group increased as compared with the DP-0 group. Besides, there were no significant differences of OHC among all groups. Similar results were reported in previous research, which pointed out that plasma exposure can improve WHC through increasing specific surface area (28).

To understand the mechanism of W/OHC changes, the assays of morphologies and specific surface area were carried out. The surface morphologies results (Figure 3) indicated that plasma treatment of 60 s exerted a “clean” effect on the surface of RPA, of which displayed a smoother surface than that in the DP-0 group. Then, the roughness of RPA in the DP-120 and DP-180 groups increased. As many similar cases discussed previously, the decrease in film roughness may be attributed to shadowing effects. In this theory, the reactive particles in the plasma system displayed a lower flux in the valley of the RPA surface (29–31). As a result, lower plasma exposure dose would present the decreasing effect of the roughness of materials. The results of specific surface area analysis (Table 2) showed that the BET specific surface area of RPA samples in the DP-120 and DP-180 groups was extremely increased as compared with the DP-0 group, while the values of that in the DP-60 group were noticeably reduced. The result was related to the structural changes in SEM analysis, possibly due to the fact that plasma treatment for 60 s reduced the roughness by destroying hydrogen and other non-Covalent bonds between the organic macromolecules of RPA, and the plasma treatment of 120 and 180 s increased the roughness of RPA by ion bombardment (32). Besides, the pore diameter analysis (Figure 4) showed that a longer time of plasma treatment can result in smaller pores with an exposure dose-dependent characteristic (33). This was possibly due to the fact that continuously plasma exposure can produce dense energetic particles, that have a high possibility of reacting with RPA samples (34, 35).

To further unravel the possible mechanism of structure changes, the experiments of FTIR and XPS were successively conducted. Four RPA samples showed similar FTIR spectra (Figure 5), while there was a slight shift from 3,427 cm^−1^ (DP-0 group) to 3,445 cm^−1^ (DP-120 group) and 3,439 cm^−1^ (DP-180 group), with an increased intensity, which implied that the oxidation reaction degree increased on the surface of the RPA samples over time (36, 37). In addition, the intensity of the peaks at 664 cm-1 and 577 cm-1 were diminished due to the C-H groups in the benzene ring which were oxidized to carbonyl and carboxyl groups. On the other hand, the XPS results showed that the oxygen atomic composition of RPA samples (Table 3) was increased, correlated with exposure time. The XPS survey spectra (Figure 6) indicated that there were only O 1s, N 1s, and C 1s in the RPA samples. As shown in Figures 6B,D, the content of the C-O group in RPA was decreased while the ratio of the C=O group was increased. As mentioned in a previous article, the C-O-H groups were oxidized to O=C-O-H on the aromatic nucleus, as a result of the oxidation reaction by plasma exposure (38).

To some extent, the weight loss curve can reflect the thermostability and even contents of bioactive compounds in any objects. As shown in Figure 7A, region I could be attributed to the volatilization of bound water in gallic acid, 2′-hydroxy-4′-methoxyacetophenone, and cellulose molecules. Region II was mainly related to the thermal decomposition of tannic acid, gallic acid, paeoniflorin, starch, and cellulose. While region III was related to the further disintegration of gallic acid and lignin, and the result of the conversion of cellulose into biochar (39–41). The TGA and DTG curves indicated that the decomposition temperature of RPA in the four groups was similar, while there was little difference at region I (Figure 7B). The decomposition rate of RPA in the DP-60, DP-120, and DP-180 groups was faster than that in the DP-0 group, and the DP-180 group displayed the highest rate. We speculated this might be due to the fact that plasma exposure treatment can modify the structures of small molecular substances or increase the specific surface area of RPA samples so that the inner substances can thermally degrade sooner. In addition, decomposition temperature, i.e., onset temperature (*To*), peak temperature (*Tp*), and conclusion temperature (*Tc*), of four RPA samples was analogous, which implied that DBD plasma treatment had no destruction effect on RPA (42).

In conclusion, our study indicated that DBD plasma pretreatment of 180 s can significantly improve the extraction efficiency of tannic acid (24.6%) and polysaccharide (12.0%), but decrease performance on paeoniflorin (2.1%) and gallic acid (5.4%). Besides, DBD plasma exposure for 60 s, 120 s, and 180 s showed no significant changes on benzoylpaeoniflorin and 2′-hydroxy-4′-methoxyacetophenone. We further found that DBD plasma pretreatment modified the extraction efficiency potentially through etching the surface and grafting polymerization by reactive species, which changed the WHC and OHC of RPA. These findings can advance our thinking about applying HVACP technology for bioactive properties enrichment and Chinese traditional medicine storage.

## Data Availability Statement

The original contributions presented in the study are included in the article/supplementary material, further inquiries can be directed to the corresponding author/s.

## Author Contributions

TJ and ZZ designed and conceived the research and drafted the manuscript. TJ, ZZ, JZ, and WO analyzed the data and interpreted the results. ZW reviewed and edited the final manuscript. All authors contributed to the article and approved the submitted version.

## Funding

The Key R&D plan of Anhui Province, under Grant No. 201904a07020013.

## Conflict of Interest

The authors declare that the research was conducted in the absence of any commercial or financial relationships that could be construed as a potential conflict of interest.

## Publisher's Note

All claims expressed in this article are solely those of the authors and do not necessarily represent those of their affiliated organizations, or those of the publisher, the editors and the reviewers. Any product that may be evaluated in this article, or claim that may be made by its manufacturer, is not guaranteed or endorsed by the publisher.
